# Bayesian approach for maize yield response to plant density from both agronomic and economic viewpoints in North America

**DOI:** 10.1038/s41598-020-72693-1

**Published:** 2020-09-29

**Authors:** Josefina Lacasa, Adam Gaspar, Mark Hinds, Sampath Jayasinghege Don, Dan Berning, Ignacio A. Ciampitti

**Affiliations:** 1 Department of Agronomy, Kansas State University, 2004 Throckmorton Plant Science Center, Manhattan, KS 66506 USA; 2 Corteva Agriscience, 7100 NW, 62nd Ave., Johnston, IA 50131 USA

**Keywords:** Plant sciences, Plant ecology, Plant physiology

## Abstract

Targeting the right agronomic optimum plant density (AOPD) for maize (*Zea mays* L.) is a critical management decision, but even more when the seed cost and grain selling price are accounted for, i.e. economic OPD (EOPD). From the perspective of improving those estimates, past studies have focused on utilizing a Frequentist (classical) approach for obtaining single-point estimates for the yield-density models. Alternative analysis models such as Bayesian computational methods can provide more reliable estimation for AOPD, EOPD and yield at those optimal densities and better quantify the scope of uncertainty and variability that may be in the data. Thus, the aims of this research were to (i) quantify AOPD, EOPD and yield at those plant densities, (ii) obtain and compare clusters of yield-density for different attainable yields and latitudes, and (iii) characterize their influence on EOPD variability under different economic scenarios, i.e. seed cost to corn price ratios. Maize hybrid by seeding rate trials were conducted in 24 US states from 2010 to 2019, in at least one county per state. This study identified common yield-density response curves as well as plant density and yield optimums for 460 site-years. Locations below 40.5 N latitude showed a positive relationship between AOPD and maximum yield, in parallel to the high potential level of productivity. At these latitudes, EOPD depended mostly on the maximum attainable yield. For the northern latitudes, EOPD was not only dependent on the attainable yield but on the cost:price ratio, with high ratios favoring reductions in EOPD at similar yields. A significant contribution from the Bayesian method was realizing that the variability of the estimators for AOPD is sometimes greater than the adjustment accounting for seed cost. Our results point at the differential response across latitudes and commercial relative maturity, as well as the significant uncertainty in the prediction of AOPD, relative to the economic value of the crop and the seed cost adjustments.

## Introduction

Maize yield has increased over the last several decades as a product of improved genetics and agronomic management practices^1,2^, as the outcome of the complex genotype by environment by management (GxExM) interaction. From a breeding perspective, improved stress tolerance has been highlighted as one of the more relevant traits for the over-time yield gain process in maize^1–4^. In this definition, ‘stress’ refers to any factor that reduces the capture or use of one or more growth resources, affecting individual plant’s growth, which is closely related to kernel set^5–7^. Newer cultivars are more efficient at setting kernels and reducing bareness at low resource availability, under stress conditions ^8–10^. Therefore, increases in yield over time are explained by the number of plants m^−2^ and the efficiency at setting and maintaining kernels at higher plant densities^11^.

Targeting the right plant density for a certain environment is a crucial factor to maximize yield for that given environment. Most grain crops have an asymptotic relationship of yield and plant density, but the sink capacity of maize ears is reduced when reaching supra-optimal densities due to their axillary position in the plant^11–13^, increasing intraspecific competition, reducing the available resources per plant, and in some situations increasing plant barrenness^12^. Plant density below the optimal will have more resources per plant with greater individual plant growth but in a population-scale the overall yield will be lower than when the canopy is at the optimal number of plants, maximizing light capture and canopy growth.

The above-mentioned aspects of maize physiology explain a better fit of quadratic functions for response curves (Eq. 1), where yield reaches an optimum at a certain agronomic optimum plant density (AOPD)^14^. A general model for this type of response can be written out as

1 $$Yield = b_{0} + b_{1} \cdot plant~density + b_{2} \cdot \left( {plant~density} \right)^{2} ,$$

where b_0_, b_1_ and b_2_ are the coefficients of the model and AOPD is directly derived from them. In order to obtain the theoretical shape of the curve, ‘b_2_′ needs to be negative. The AOPD is modified by (i) the relative maturity of the maize hybrid, inversely related^7^, (ii) sowing date^6,15–17^, (iii) water supply^18,19^, (iv) nutrient availability^20^ and (v) pest management^21,22^. Short-cycle hybrids produce smaller plants and need greater stand density for resource capture^7,23,24^. The rest of the above-mentioned factors are related to resource offer for plant growth. Several of those factors are closely entangled and their effects are hard to single-out. Most studies summarize this pool in the relationship between maximum yield and AOPD^20^. Environments can be characterized by their climate, soil characteristics and latitude to make inferences about growth conditions^25^. However, there are yearly variations (e.g. radiation, rainfall and temperature) that may produce different responses to density for the same hybrid^18^ and require different management decisions.

From the statistical standpoint, the yield-density response curves are commonly addressed using a Frequentist approach. In addition, the normal distribution has been the main assumption for the parameters of the function. This might not always be true, especially for a quadratic response curve with a negative coefficient for the quadratic component (‘b_2_′ in Eq. 1). Although this problem might be tackled with more sophisticated modeling from a Frequentist approach, inferences are still limited due to their theoretical framework^26^. Moreover, there is loss of information regarding variability of the estimators of the model parameters. Bayesian statistical methods retrieve the entire posterior distribution and allow making conclusive inferences. These methods can be utilized more often nowadays due to increasing computational power^27^. There is a growing area among statisticians developing software to implement Bayesian modeling^28^ and many fields in the biological sciences are starting to implement them^29^.

Lastly, targeting the right economic optimum plant density (EOPD) is a realistic production approach^30^. Maize is sold as a commodity, where farmers are price-takers. Then, the price of the grain is the same as the marginal benefit the farmer receives for selling one extra unit. Seed costs have been increasing over the years, having a relatively inelastic demand. In addition to studying yield stability to weather, producers should perform sensitivity analyses to support their decisions.

This study included a dataset of maize hybrid by seeding rate experiments conducted from 2010 to 2019, as well as seed and grain prices from 2010 to 2018. Thus, the aims of this research were to (i) quantify AOPD, EOPD and yield at those plant densities, (ii) compare and obtain clusters of yield-density for different attainable yields, and (iii) characterize their influence on EOPD variability under different economic scenarios, seed cost to corn price ratios.

## Results

### Bayes agronomic optimum plant density (AOPD) and latitude effect

**Figure 1:**
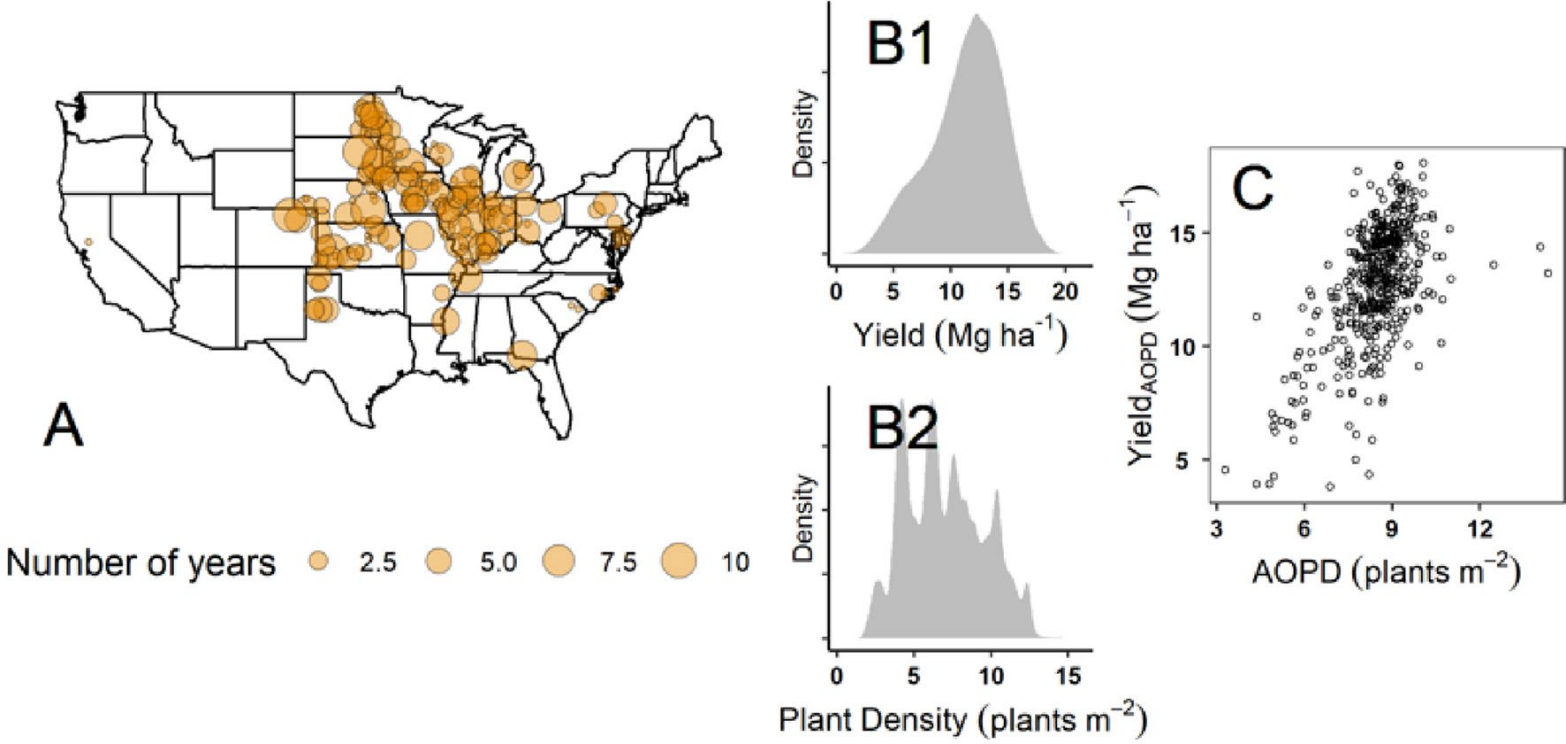
Data description. (**A**) Geographical datapoint distribution within the continental US territory (the size of the bubbles represents the number of years). This map was created with the ggplot2 package^33^ in R^34^; (**B1**) Yield distribution (raw data); (**B2**) Plant density distribution (raw data); and (**C**) Relationship between Bayes estimated agronomic optimum plant density (AOPD) and yield_AOPD_ for each site-year of the dataset ranging from 2011–2019 period.

A posterior predictive check showed that the values generated by the model were consistent with the original data^31^. Convergence diagnostics for all the variables were acceptable, according to established standards: R_hat_ values were 1 for all the considered variables^32^. There was a positive relationship of estimated AOPD and yield_AOPD_ (Fig. 1C).

**Figure 2:**
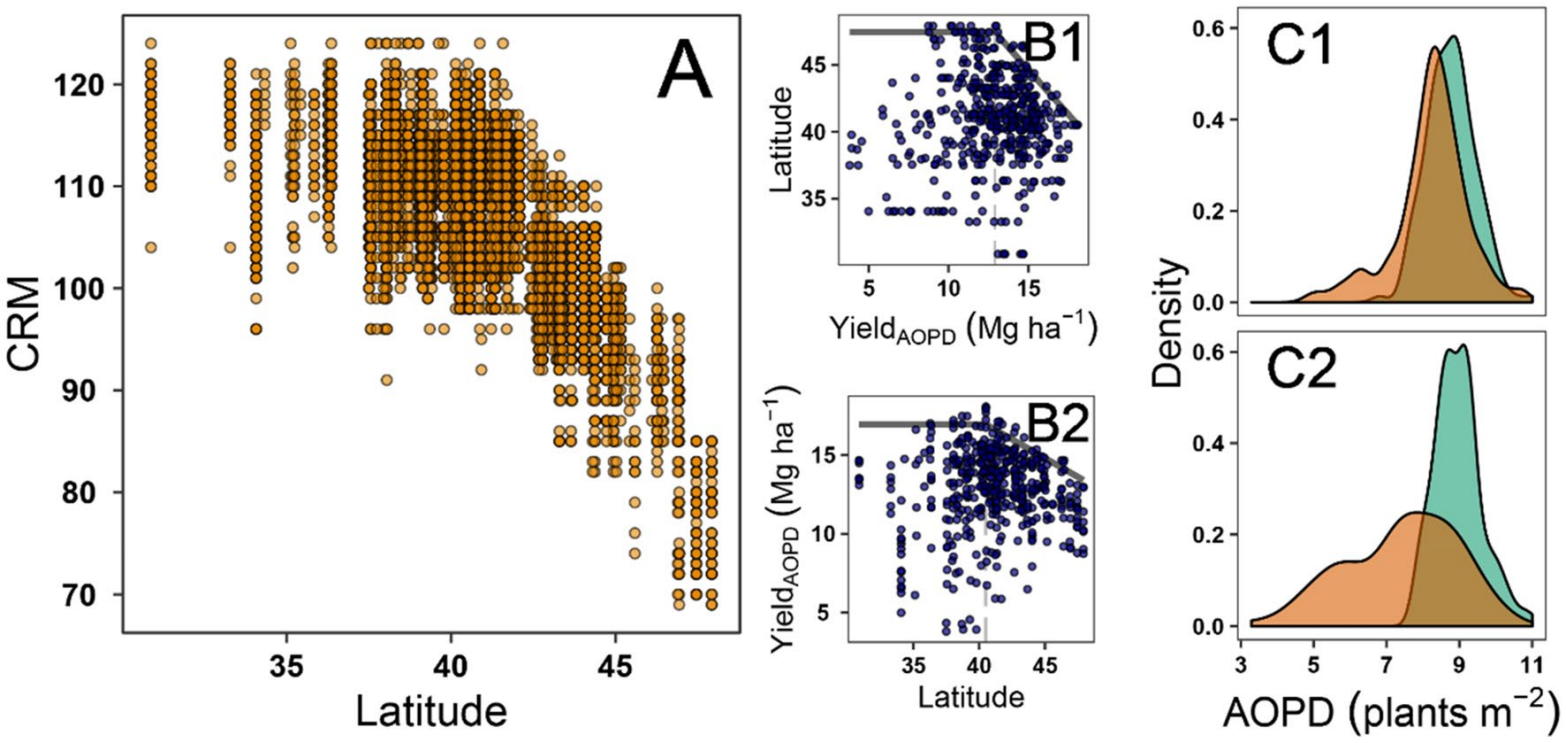
Yield-latitude relationships. (**A**) Relationship between commercial relative maturity (CRM) and latitude. (**B**) Relationship between latitude and yield_AOPD_ (**B1**), and between yield_AOPD_ and latitude (**B2**), (**C**) AOPD distribution for high and low yield_AOPD_ at locations North (**C1**) and South (**C2**) of 40.5 N.

A threshold for different responses to plant density was found at 40.5 N, with maize hybrid CRM rapidly declining for higher values (northern latitudes) based on the dataset (Fig. 2A) and as a result of the for the upper boundary (99 quantile) linear-plateau regression of the yield_AOPD_ as a function of latitude (Fig. 2B2). From herein onwards, the group of locations below 40.5 N will be named as group ‘I’, whereas the group of locations above that latitude as group ‘II’. The inverse function showed that yield_AOPD_ plateaued at 12.9 Mg ha^−1^. The AOPD presented a contrasting distribution for low- versus high-yielding locations (*i.e.* lower (orange) or higher (blue) than 12.9 Mg ha^−1^, respectively) only in group I (Fig. 2C2). The AOPD portrayed similar distributions for both yield environments for the group II (Fig. 2C1).

Optimal number of clusters for the southern latitudes (below 40.5 N) was 4, and 5 for the northern latitudes (above 40.5 N). The AOPD and yield_AOPD_ for these groups were described with high density intervals (HDI) of 80% (Table 1); some clusters had a bimodal distribution (i.e., cluster 1 and 3 for the groups I and II) of one of their variables, and then include more than one sub-interval for their HDI estimation^35^.

**Table 1:** High density intervals (HDI) of 80% percent for the agronomic optimum plant density (AOPD) and maize yield at the AOPD for each cluster within each group, group I—southern latitudes and group II—northern latitudes.

Group	Cluster	AOPD (plants m^−2^)		Yield_AOPD_ (Mg ha^−1^)	
		Lower boundary	Upper boundary	Lower boundary	Upper boundary
I Southern (below 40.5 N)	1	3.8	4.9	4.3	7.0
		10.6	12.5	7.1	8.3
	2	9.2	10.3	6.9	9.6
	3	10.6	12.5	8.4	9.8
		13.2	14.8	8.0	9.4
	4	14.9	16.5	8.4	9.8
		16.6	17.0	7.1	8.3
II Northern (above 40.5 N)	1	5.4	5.8	7.9	9.9
		6.6	9.2		
	2	7.6	9.1	10.8	12.1
	3	7.5	9.6	12.3	13.4
				13.5	13.6
	4	8.1	9.6	13.9	15.3
	5	8.2	9.8	15.7	17.7

Notice that clusters are named in increasing order according to the yield distribution. Clusters with two lower and upper boundaries presented a bimodal distribution of the AOPD factor.

**Figure 3:**
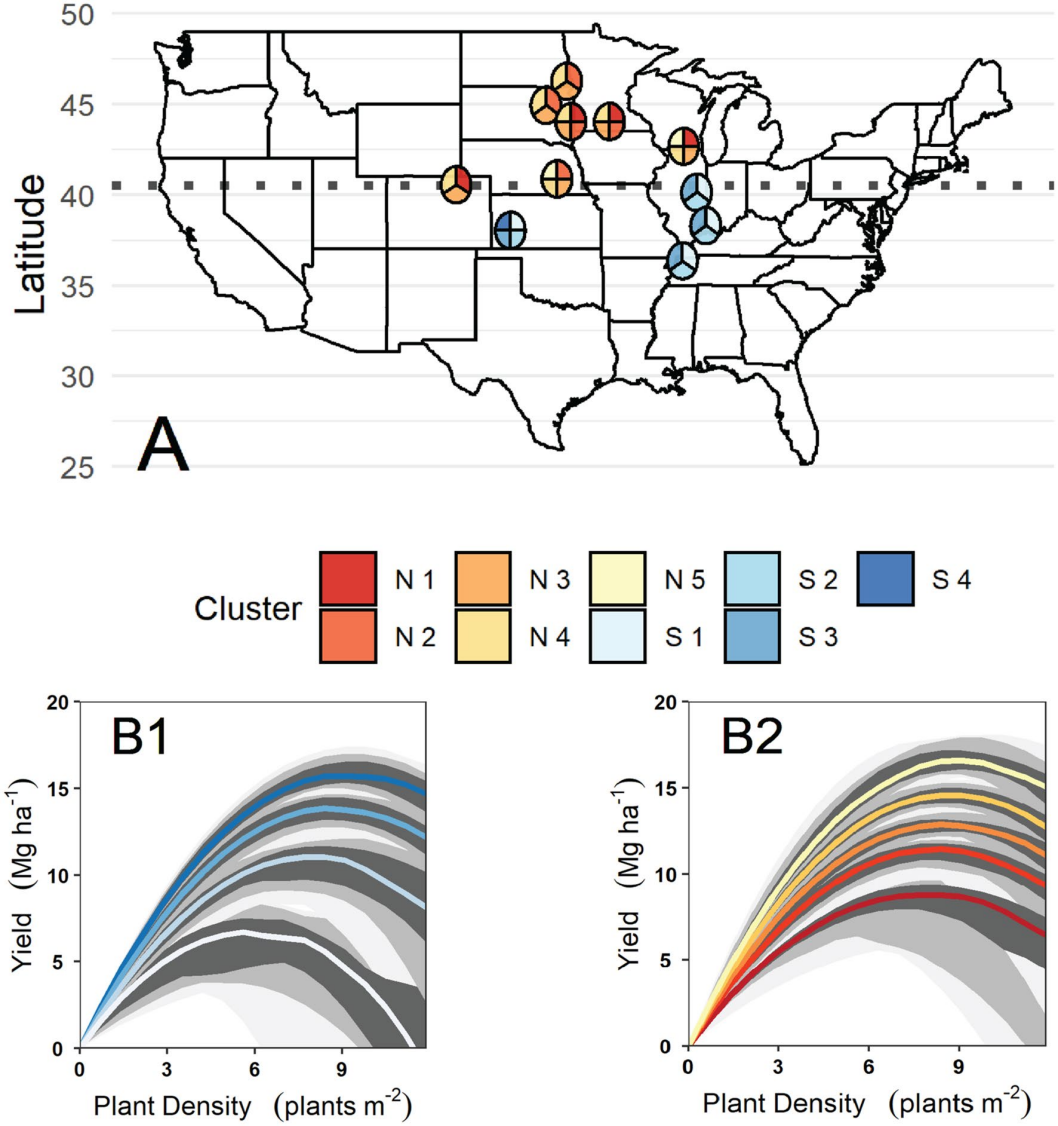
Cluster geographic distribution. (**A**) Cluster frequency for locations with more than 7 observed years. This map was created with the ggplot2 package^33^ in R^34^; (**B**) Yield response to plant density for: (**B1**) Group I (S): Southern latitudes (i.e., below 40.5 N), (**B2**) Group II (N): Northern latitudes (above 40.5 N). Clusters are numbered in increasing order, according to their yield distribution.

Considering all site-years, the highest-yielding clusters were more frequent around 40.5 N, i.e. northernmost section of Group I and southernmost section of Group II. A largest proportion of the locations yielding higher than 14 Mg ha^−1^ were found between 39 and 42.5 N (75% of the cases, Fig. 2B2). These locations were included in the clusters with the largest response for the yield-density models. In contrast, low-yielding scenarios were found towards the southern locations, in environments more prone to drought and heat stress conditions. An exception to that last statement can be mentioned for irrigated trials, e.g. field studies located in Texas and Kansas, that corresponded to the most responsive clusters (Fig. 3A).

In order to obtain a probability of the distribution for each yield-density model within each location, only sites with more than 7 years of data, with the goal of sampling more weather variation, were considered in this last step of the analysis. A total of 4 environments were retained for the southern latitudes and 7 for the northern latitudes (Fig. 3A) to obtain the relative frequency of each yield-density response model. More responsive clusters were largely located at latitudes around 40.5, as above mentioned. Each location had temporal variability regarding the clusters. When dissecting each great group into 3 latitude strips, differences in the response curves could still be distinguished. In II, higher latitudes include high-yielding site-years with less frequency and the highest-yielding cluster is absent above 44 N.

Group I showed a positive relationship between AOPD and yield, which was scarcely the case for group II. High-yielding scenarios normally require greater seeding rates, this positive relationship was true for locations below the 40.5 latitude, where AOPD level is mainly driven by the attainable yield (Fig. 3A). However, this relationship was less driven by attainable yields at the northern latitudes, presenting a narrower variation in AOPD (7–9.8 pl m^−2^, 90% of the data points) for the yield-density models relative to the one observed (5.5–10.1 pl m^−2^, 90% of the data points) for the southern latitudes (Fig. 3B).

### Economic optimal plant density (EOPD)

**Figure 4:**
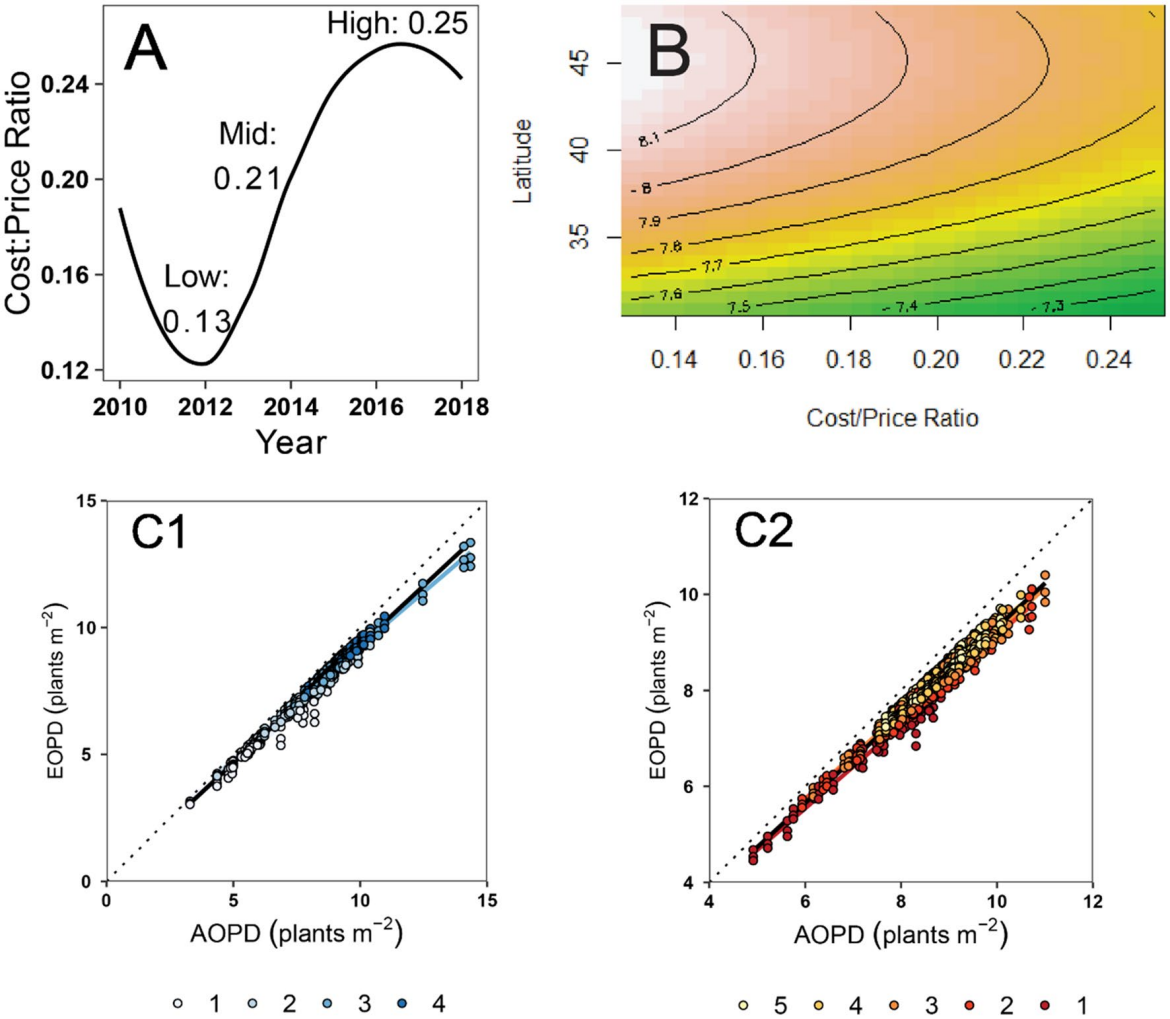
(**A**) Cost/price ratio evolution through the years for the US. (**B**) EOPD variation at different latitude and price/cost ratio combinations. (**C**) EOPD vs. AOPD regression for (**C1**) Group I (S) and (**C2**) Group II (N); dashed lines represent the 1:1 relationship.

The gap between AOPD and EOPD varied by economic scenario and the shape of the response curve for yield to plant density. The latitude (nested with CRM) factor reflected also as attainable yield is the main factor driving the changes in the EOPD, with the EOPD decreasing with the latitude from > 40 N to < 35 N (Fig. 4B). As a secondary but still relevant factor for the northern latitudes (above 40.5 N), the EOPD substantially decreased (i.e., 81,000–78,000 pl ha^−1^) with the cost:price seed ratio (from 0.14 to 0.24 units). Thus, for the northern latitudes, EOPD is more dependent on the economic scenario than for the southern latitudes.

A standardized major axis (SMA) regression of EOPD versus AOPD showed that the slopes are significantly different between group I and II, as well as between clusters in a certain latitude group (Fig. 4C1,C2, Supplementary Table 1). Although independent from the division between group I and II, this analysis confirms a different response for the northern latitudes. The relationship between AOPD and EOPD was significantly different between groups I and II or between clusters within a group (i.e. the slope of the regression was significantly different from 1). Considering that the slopes are less than 1 for this regression, the higher the AOPD, the larger is the gap between both AOPD and EOPD, with this last economic optimum reduced more than proportionally relative to the maximum agronomical value. However, the absolute value of the plant density reduction is similar to the uncertainty there is in AOPD.

## Discussion

This study presents a novel Bayesian approach for a uniquely large dataset spanning from 2010–2018 period including several US counties for variation in both AOPD and EOPD within and across sites. A more regional-specific yield-density model was obtained based on retrieving not only the AOPD and yield at AOPD for each site-year but by obtaining the distribution of each factor and considering the effect of latitude (nested with CRM) and the attainable yields. Even though we tested a large dataset, we do acknowledge the lack of focus on the hybrid effect due to the intrinsic complexity of this factor^36,37^, genetic gain over time and changes in hybrids within and across locations and time. However, we decided to focus on the larger picture in order to understand the changes in plant density at different geographical locations, attainable yields, and by sampling the intrinsic weather variation observed in the last seven years of US maize production.

The refinements tested in this research by using Bayesian, rather than a Frequentist approach, permitted an improved study of the distribution and obtained better inferences for each trait of interest^38^. A Frequentist analysis would normally produce a single-point estimator (e.g. maximum likelihood estimator), presenting an intrinsic variability sometimes reported, but usually not taken into consideration for decisions or comparisons between response curves^25^. Bayesian methods yielded the entire posterior distributions instead, portraying for this study that the variability in the estimation is greater than the suggested reduction accounting for seed cost. Decision makers should be aware of this variability considering a probability analysis for their decision-making process. In a nutshell, the results of this analysis may be reduced to a single-point recommendation (e.g. the mean or the mode), but the posterior distribution of AOPD represents all the likely values this parameter might take.

A second lesson, consistent with a past study looking at yield-density relationship^39^, is that latitude and hybrid relative maturity (CRM) in combination with the attainable yields within this factor, play a critical role in identifying more regional specific yield-density models. Similar responses on yield to plant density were more recently documented in maize crop^17,25,30,39–42^, studying individual factors, in public- and private- field research plots and on-farm experimentation. The overall optimal plant density values reported in the scientific literature presented similar variation to the uncertainty ranges documented in this study. In many scenarios those variations were primarily related to factors such soil moisture, N conditions, and weather among other factors^37^, with plant density increasing with attainable yields until an optimal point. In the current study, high-yielding environments (in larger proportion for latitudes below 40.5 N), AOPD presented a stronger relationship with attainable yield in part due to the amount of available resources potentially reducing the crowding stress, supporting a greater number of plants per unit of area. This trend agrees with previous studies comparing density responses to different abiotic stresses^43,44^. On the contrary, northern latitudes (above 40.5 N) depicted less variation in AOPD even under changes in attainable yields. For these environments, the larger relevancy of short-season hybrids connected with usually later planting dates^17^ relative to the southern locations will further restrict the extension of the growth cycle and potentially compromised light captured at canopy-scale when plant density is not at the optimal level^7,16,45^. Less time to flowering will (i) reduce biomass at silking (i.e. less biomass partitioned to the roots for water and nutrient uptake) and (ii) reduce in-depth root exploration. To sum up, there is a tradeoff between the capacity to capture resources (e.g. leaf area for light interception) and the level of intraspecific competition^23^.

Contrary to our expectation, the AOPD values to maximize yield differed with the EOPD to increase profits (by reducing seed cost), but only in relatively small amounts. Those adjustments for EOPD were within the variation documented to estimate AOPD—in this case reported applying Bayesian statistics. Previous studies found no statistical significance regarding variable seeding rate^40^, giving a hint about the complexity in the estimation of economic optima when the prediction of the uncertainty can mask this difference. Still, farmers should not overlook the EOPD but take into consideration the uncertainty in the estimation of these relevant parameters. Economic scenarios, reflected in changes over time in the cost:price ratio, have more impact on seeding rate decisions for northern latitudes while the attainable yield in each location was a key factor regardless the geography. Past studies reflected the potential benefits of using an EOPD for variable rate seeding as standard practice for improve profits^20,41,46^.

Future studies should look at the differential fertilizer costs which could increase variable costs and thus further impact EOPD estimation, more in high-yielding environments^20,42^. In addition, different hybrids have different responses to plant density^7,37^ and nitrogen availability^47^. These factors were not included in the current model due to computing limitation and more precisely the fact that hybrids were not repeated every year. Hybrids with different levels of drought tolerance may show very different responses when stressed^18,48^. This new perspective might help gain insights about hybrid variability and whether EOPD is much dependent on individual hybrids as well. Lastly, more guidance on improving our predictions on yield-plant density relationships not only based on hybrid (genetic yield potential) but even more relevant on the interaction between the right management for the specific soil and weather combinations for that environment, ultimately increasing the ability of the growers for having a probability to assess the risk of selecting the right seeding rate for their fields.

## Conclusions

This study identified common yield-density response curves as well plant density and yield optimums for 460 site-years. Site-years with greater attainable yield presented more responsive yield-density curves, more concentrated around 40.5 N latitude. Locations below 40.5 N showed a positive relationship between AOPD and maximum yield, in parallel to the higher potential level of productivity. Both latitude and hybrid relative maturity CRM factors were highly correlated for the northern locations and thus hard to detangle on their effect in AOPD and yield_AOPD_ for those geographical locations. Lastly, for those sites, EOPD was not only dependent on the final attainable yield but on the cost:price ratio, with high ratios favoring reductions in EOPD at comparable attainable yield levels. Overall hybrid effect and consideration of other variable costs should be considered for further research studies. Yet our results provide the foundation for proper data evaluation, including a measure of the uncertainty for the estimations of AOPD, yield at AOPD, and consequently, EOPD, and providing more reliable estimates to improve the complex farming process of selecting the right seeding rate for each environment.

## Methods

Maize hybrid by seeding rate trials were conducted in 24 states in the US from 2010 to 2019 in at least one county per state. The experimental design for trials was a randomized complete block design (RCBD) in a split-plot arrangement. Plant density was the whole plot treatment and hybrids were in the subplot level. There were five target plant densities: 44,475; 64,247; 84,016; 103,784 and 123,553 plants ha^−1^ from 2011-to-2019. The study was conducted in research sites and farmer fields with plot size of 3.05 m (4-rows) wide by 5.4 m long in 0.76 m row spacing, and there were 2-to-5 replicates at each site. Plots were consistently fertilized with all recommended nutrients for their respective location and related to their attainable yield levels. Only a few (five) trials were irrigated in specific years in one county each of Texas, Nebraska, Kansas, and Mississippi.

Maize hybrid comparative relative maturity (CRM) ratings were obtained from Corteva Agriscience. Further details related to the ratings can be found at Pioneer.com^49^. Each county has the recommended CRMs for that location, and hence the genotypes differed across latitude. At all locations, yield was recorded on the central two rows in each plot, grain moisture was measured, and yields adjusted to 155 g kg^−1^. Frequency distribution for all hybrids (Fig. 1a), data by plant density (Fig. 1b) and yield (Fig. 1c) relative to hybrid release year were explored.

### Model fitting

A Bayesian hierarchical quadratic response model was fitted to the data with the following priors:

2 $$y_{ij} \sim Normal\left( {\mu_{j} , \sigma_{j}^{2} } \right);$$

3 $$\mu_{ij} = b_{0j} + b_{1j} \cdot x_{ij} - b_{2j} \cdot x_{i}^{2} ;$$

4 $$b_{0j} \sim gamma\left( {1,1} \right);$$

5 $$b_{1j} \sim normal\left( {0.08,0.5} \right);$$

6 $$b_{2j} \sim gamma\left( {1,1} \right);$$

7 $$\sigma^{2} \sim gamma\left( {1,1} \right).$$

where $y_{ij}$ is the yield (kg ha^−1^) in observation *i* and site-year *j*, $\mu_{j}$ and $\sigma_{j}^{2}$ are the mean and the variance of the site-year *j*. Observations were comprised by several hybrids and plant densities at 460 site-years, with a total of 118,273 data points. Site-years were considered independently because the dataset did not include all the locations throughout the years. The AOPD and the yield at AOPD were estimated from the posterior distribution. The EOPD was estimated for three different cost/price ratios: 0.14, 0.21 and 0.27. These ratios refer to the means for the years 2011 to 2013, 2014 to 2016, and 2016–2018, respectively. The Bayesian hierarchical models were fitted to the data using Stan^28^ using R software^34^. The R_hat_ values were used to check for correct mixing and convergence of the chains (a value of 1 means they reached convergence)^32^.

Across all maize hybrids within a location and for each site-year (Fig. 1A), AOPD level and yield at AOPD was gathered for further evaluation and analysis (Fig. 1C).

### Clustering

Locations were divided into two latitude groups with a threshold of 40.5 N. This value was obtained from a 95-percentile linear-plateau regression between yield and latitude (Fig. 2). Clusters of common responses to plant density were identified using Euclidean distance and the *k-means* method and the *silhouette* index to determine the best number of clusters^50^. A restriction of maximum eight clusters within each group was selected to obtain interpretable results. Median values of Yield_AOPD_ and *b*_*1*_ ([3]) were used as the clustering variables.

### Economic analysis

Grain prices from 2010–2018 were obtained from USDA-NASS^51^ and seed prices were provided from Corteva Agriscience for the same time period. Cost/Price Ratio was calculated for each moment as

8 $$\frac{C}{P} = \frac{{Seed~Cost_{i} \left( {USD \cdot seed^{ - 1} } \right)}}{{Grain~Price_{i} \left( {USD \cdot Mg_{grain}^{ - 1} } \right)}},$$

where *i* is the month of the year (ranging from January 2010 to December 2018). This data was then divided into three groups, representing possible price scenarios: low, mid and high (Fig. 4A). Then, the EOPD was estimated for each scenario as the value of plant density for which the slope of the response function equals that ratio. This outcome was put into a response surface model^52^ to analyze the sensitivity of EOPD at varying cost/price ratio and latitude combinations.

Shipping and selling costs were not take into account in order to simplify the analysis.

## Supplementary information

Supplementary file 1
