# Supplementary material for: Bayesian approach for maize yield response to plant density from both agronomic and economic viewpoints in North America
Source: Sci Rep. 2020 Sep 29;10:15948. doi: 10.1038/s41598-020-72693-1 (PMC7525453; doi:10.1038/s41598-020-72693-1)
Supplement: Supplementary file 1 [file 41598_2020_72693_MOESM1_ESM.docx]

**Bayesian approach for maize yield response to plant density from both agronomic and economic viewpoints in North America**

Josefina Lacasa^1^*, Adam Gaspar^2^, Mark Hinds^2^, Sampath Jayasinghege Don^2^, Dan Berning^2^, and Ignacio A. Ciampitti^1^*

^1^Department of Agronomy, Kansas State University, 2004 Throckmorton Plant Science Center, Manhattan, Kansas, 66506, US.

^2^Corteva Agriscience, 7100 NW, 62nd Ave., Johnston, IA, 50131, US.

Correspondence and requests for materials should be addressed to

J.L. (email: [lacasa@ksu.edu](mailto:lacasa@ksu.edu)) or I.A.C. (email: [ciampitti@ksu.edu](mailto:ciampitti@ksu.edu))

**Supplementary information**

**Supplementary Table 1.** Standard major axis (SMA) regression coefficients for each cluster, with the economic optimum plant density (EOPD) as a function of the agronomic optimum plant density (AOPD) for each latitude group.

| Group | Cluster | Slope | p-value |
| --- | --- | --- | --- |
| I | 1 | 0.91 | < 2.22e-16 |
|  | 2 | 0.93 | < 2.22e-16 |
|  | 3 | 0.93 | < 2.22e-16 |
|  | 4 | 0.94 | < 2.22e-16 |
|  | 5 | 0.95 | < 2.22e-16 |
| II | 1 | 0.93 | < 2.22e-16 |
|  | 2 | 0.93 | < 2.22e-16 |
|  | 3 | 0.94 | < 2.22e-16 |
|  | 4 | 0.90 | < 2.22e-16 |
